# Women in sports: the applicability of common national reference intervals for inflammatory and coagulation biomarkers (HemSter Study)

**DOI:** 10.11613/BM.2021.010702

**Published:** 2020-12-15

**Authors:** Vanja Radišić Biljak, Valentina Vidranski, Lana Ružić, Ana-Maria Simundic, Tihomir Vidranski

**Affiliations:** 1 Department of Medical Laboratory Diagnostics, University Hospital „Sveti Duh“, Zagreb, Croatia; 2 Department on Oncology and Nuclear Medicine, University Hospital Center “Sestre milosrdnice”, Zagreb, Croatia; 3 Department of Sport and Exercise Medicine, Faculty of Kinesiology, University of Zagreb, Zagreb, Croatia; 4 Faculty of Pharmacy and Biochemistry, University of Zagreb, Zagreb, Croatia; 5 Department of Kinesiology, Faculty of Education, Josip Juraj Strossmayer University of Osijek, Osijek, Croatia

**Keywords:** sports, international physical activity questionnaire short form (IPAQ-SF), inflammation, blood coagulation, reference ranges

## Abstract

**Introduction:**

Intensive physical activity causes functional and metabolic changes in the athlete’s organism. The study aimed to verify the common national available reference intervals (RIs) for common inflammatory and screening coagulation tests in a population of healthy young female athletes.

**Materials and methods:**

One hundred and twenty-one female athletes (age range: 16–34), from various sports disciplines (water polo, handball, volleyball, football, basketball), were included in the study. All participants completed the international physical activity short-form questionnaire. Blood samples were collected between 8–10 am, after an overnight fast, before any physical activity. Reference intervals were determined according to Clinical & Laboratory Standards Institute EP28-A3C Guidelines.

**Results:**

Calculated RIs for white blood cell count (WBC), prothrombin time (PT), and activated partial thromboplastin time (APTT) ratio were in accordance with the common national RIs. Calculated RI for C-reactive protein (CRP) was lower (< 2.9 mg/L) than the proposed cut-off for a healthy population (< 5.0 mg/L). Reference interval for fibrinogen was higher (1.9–4.4 g/L), than the available RIs (1.8–3.5 g/L). D-dimer cut-off value was set at 852 µg/L fibrinogen equivalent units (FEU), higher than the proposed 500 µg/L FEU for venous thromboembolism (VTE) exclusion.

**Conclusions:**

The applicability of the available RIs for WBC count, PT, and APTT-ratio was confirmed. However, RIs for CRP and fibrinogen differed significantly than the available common national RIs for the healthy non-athletes’ population. A higher cut-off for D-dimers should be extensively verified before implementation for VTE diagnosis exclusion in a group of healthy young female athletes.

## Introduction

Inflammation and coagulation constitute two host defence systems with complementary roles in eliminating invading pathogens, limiting tissue damage, and restoring homeostasis (*1*). There is an increasing body of evidence that extensive cross-talk between systems of inflammation and coagulation exists, where inflammation leads to activation of coagulation and, consequently, coagulation also markedly affects inflammatory activity (*2*). The intensive physical activity causes significant functional and metabolic changes and adaptations in the athlete’s organism (*3*, *4*).

It was well documented that the immune system is very responsive to exercise, with evidence on anti-inflammatory, as well as on pro-inflammatory effects of exercise (*5*). Although regular exercise offers protection against all-cause mortality and is associated with a reduced incidence of infection compared to a sedentary state, the athletes engaged in heavy training programs appear to be more susceptible to infection, with females being at greater risk than males (*4*, *6*-*8*). High exercise training workloads are linked to immune dysfunction and inflammation (*4*).

The activation of haemostasis depends on the intensity of the exercise (*4*). Athletes are at greater risk of developing a thrombotic event, as strenuous exercise may pose a significant risk factor, especially if combined with exposure to several other risk factors such as travel, trauma, immobilization, or hemoconcentration (*4*, *9*, *10*). The intensive exercise was previously proven to be associated with enhanced platelet aggregation, leucocytosis, the elevation of tissue-plasminogen activator levels, activation of the fibrinolytic system, and a decreased ability of the blood to clot and generate thrombin (*11*, *12*).

There is also some evidence that the inflammatory and coagulation changes after strenuous physical activity may occur in athletes, with some indication that, at least white blood cell count (WBC) returns to pre-existing values within 3-24h (*7*, *13*).

From a clinician’s perspective, it seems that there is a lack of steady-state controlled studies which would enable to analyse possible differences in inflammatory and coagulation biomarkers in athletes at rest. As each athlete might become a patient at some point, his/her laboratory results would only be compared with the reference intervals (RIs) that are applicable for the general population (from which those had been derived). For that reason, the hypothesis was set that due to the specific changes in inflammatory and coagulation biomarkers, as a result of metabolic adaptations in an athlete’s body, the general RIs would not be applicable. This might lead to severe post-analytical error, interpreting some results falsely as pathological, when they are actually normal, and lead to further unnecessary tests, delayed diagnosis, and consequently patient anxiety and harm.

To better understand how physical activity and subsequent changes in various biomarkers affect the applicability of general RIs in athletes, a large multicentre study entitled “Women in sports: applicability of available reference intervals (HemSter Study)“ was launched in 2019. The study included four separate goals, one of which has already been successfully addressed (*14*). The aim of this study was to assess the applicability of the common national available RIs for common inflammatory and screening coagulation tests in a population of healthy young female athletes, in the fasting state, prior to any physical activity.

The RIs for stated biomarkers are derived from a national harmonization project and are obligatory for all medical-biochemistry laboratories (MBLs) in Croatia (*15*). While these RIs are valid for a general population, they may not be applicable for young female athletes, because significant metabolic changes and adaptations in the athlete’s body were not taken into consideration as a significant covariate. To the best of our knowledge, our study is the first such attempt.

For those biomarkers where the common national RIs (or literature cut-offs) would not be applicable, new RIs for young female athletes should be proposed.

## Materials and methods

### Subjects

One hundred and twenty-one young healthy female athletes, playing several most popular team sports (water polo, handball, volleyball, football, basketball) were included in the study. The study group was extensively described in a paper by Radišić Biljak *et al.* (*14*). The information about their menstrual cycle and the usage of oral contraceptives (OC) was collected. The habitual physical activity score for all participants was assessed by the Short Form of the International Physical Activity Questionnaire (IPAQ-SF) (*16*). All athletes were recruited in four larger Croatian cities (Zagreb, Varaždin, Čakovec, and Osijek) between February and April 2019.

The Ethical Committee of the University Hospital „Sveti Duh“ (Zagreb, Croatia) granted the approval for the study. All participants signed the informed consent form.

### Materials

By taking into account all procedures described in the European Federation of Clinical Chemistry and Laboratory Medicine (EFLM) 2019 Recommendation for venous blood sampling, blood samples were collected between 8 and 10 am, after an overnight fast, and prior to any kind of light or intense physical activity (*17*). All athletes were at resting state at least for 12 hours. To standardize the procedure, even more, all venepunctures were performed during weekdays, and prior to any competition events that are usually held in weekend days. Samples were drawn into 10 mL serum tube with clot activator, 2 mL K_2_EDTA tube for complete blood count (CBC) measurements, and 4.5 mL 3.2% sodium citrate tube for coagulation parameters (all Beckton Dickinson, USA). K_2_EDTA tubes were put on a mechanical roller for 10 minutes prior to analysis. To obtain platelet-poor plasma (platelet count < 10 x10^9^/L), coagulation tubes were centrifuged immediately for 15 minutes at 1500xg at room temperature, following the Clinical & Laboratory Standards Institute (CLSI) H21-A5 Guidelines for collection, transport, and processing of blood specimens for testing of plasma-based coagulation assays and molecular haemostasis assays (*18*). Serum tubes were kept at room temperature for 30 minutes to allow the blood to clot and then centrifuged for 10 minutes at 3000xg. All samples were analysed in duplicate within an hour after venepuncture.

### Methods

Complete blood count measurements were done on an automated 6-part differential haematology analyser Advia 2120i (Siemens, Erlangen, Germany). Coagulation biomarkers (prothrombin time (PT), activated partial thromboplastin time (aPTT), fibrinogen (Fbg), D-dimers)) were measured on BCS XP coagulation analyser (Siemens, Marburg, Germany), by the following methods: PT – photo-optical method (Dade Innovin %), APTT – photo-optical method (Dade Actin FS), Fbg – modification of the Clauss method (Multifibren U), and D-dimers – particle-enhanced immunoturbidimetric assay (Innovance D-dimer)). An aPTT-ratio was calculated according to the National recommendations for blood collection, processing, performance and reporting of results for coagulation screening assays prothrombin time, activated partial thromboplastin time, thrombin time, fibrinogen and D-dimer (*19*). The recommendations state that the aPTT ratio is calculated as the ratio of patient’s aPTT divided by the mean value of the reference interval for the particular system (reagent/coagulometer) used. For the Siemens BCS XP analyser and Actin FS reagent combination the mean value of the RI was 27.5 s, so an aPTT-ratio was calculated for each aPTT measurements in seconds as following: aPTT (s)/27.5 s. C-reactive protein measurements were done by the immunoturbidimetric method on a Beckman Coulter AU680 chemistry analyser (Beckman Coulter, Brea, CA, USA). An average quantitative measure of imprecision (coefficient of variation, CV%), obtained by long term measurements of commercial quality control samples at normal physiological concentrations, for all methods was as follows (mean value of the commercial control/CV%): WBC – 7.2 x10^9^/L/1.24%, PT – 93.1%/4.84%, aPTT – 26.5 s/2.11%, Fbg – 2.8 mg/L/5.82%, D-dimers – 330 µg/L FEU/6.58%, and CRP – 3.6 mg/L/2.76%.

### Statistical analysis

Participants were divided into subgroups according to their sport. As the majority of the subgroups were small (N < 30), all variables, except age, are given as a median and interquartile range (IQR). Age is given as median (min–max). To evaluate the homogeneity of a group, the difference between all demographics, as well as between measured variables among subgroups was tested with the Kruskal-Wallis test. To assess possible differences among the exercise subgroups determined by IPAQ-SF scores, the Kruskal-Wallis test was performed. The statistical analyses were performed using MedCalc Statistical Software version 16.2.0 (MedCalc Software, Ostend, Belgium), with a P-value of less than 0.05 considered as the threshold of significance. Reference intervals were determined according to CLSI EP28-A3C 2010 Guidelines and compared to the common national RIs, applicable in MBLs in Croatia (*15*, *20*).

## Results

Out of 121 female athletes enrolled in the HemSter study, only six were OC-users at the time when the sampling was done. Because OC could alter plasma concentrations of many components of the coagulation and fibrinolysis system, it was decided to exclude those athletes from further statistical analysis, thus remaining a total of 115 athletes (*21*). A comparison of the basic demographic characteristics of the subjects was in line with the one already published in an article by Radišić Biljak *et al.* (*14*). There were statistically significant differences in age (P = 0.046), weight (P < 0.001), height (P < 0.001), and body mass index (BMI) (P < 0.001) between female athletes across different sports, their quantitative measure of physical activity (MET) did not differ significantly among the groups (P = 0.226). All measured parameters did not differ among subgroups of various sports, which enabled merging all the data into one group for reference intervals verification and determination (Table 1). The majority of the participants (96/115, 84%) were categorized as subjects with high physical activity level. A moderate physical activity level was observed in 10% (12/115) of the athletes, while only 6% (7/115) of the athletes were classified as the low physical activity category.

**Table 1:** Comparison of basic demographic characteristics of the study participants (female athletes, N = 115)

**Female athletes** **N = 121**	**Water polo** **N = 8**	**Handball** **N = 64**	**Volleyball** **N = 24**	**Football** **N = 9**	**Basketball** **N = 10**	**P**
Age, years	22 (18–34)	21 (18–29)	20 (18–28)	17 (17–27)	22 (16–30)	0.046
Weight, kg	73 (70–80)	69 (64–75)	71 (70–76)	58 (56–64)	66 (58–70)	< 0.001
Height, cm	174.5 (170.5–179.5)	173 (168.5–176)	180 (179–185.5)	167 (164–173.5)	176.5 (169–180)	< 0.001
BMI, kg/cm^2^	24.4 (23.6–25.1)	22.6 (21.5–24.3)	21.7 (20.7–22.2)	21.0 (20.6–21.4)	21.8 (20.9–22.9)	< 0.001
MET, min	7452 (2796–8990)	5226 (4029–7175)	7980 (4572–11, 098)	3822 (1910–11,519)	6289 (4653–8586)	0.226
WBC, 10^9^/L	5.9 (5.5–6.6)	6.5 (5.6–8.0)	6.2 (5.4–7.5)	6.5 (5.9–10.2)	6.0 (4.9–7.0)	0.406
CRP, mg/L	0.40 (0.35–0.65)	0.50 (0.30–1.05)	0.40 (0.30–1.00)	1.00 (0.63–1.78)	0.50 (0.40–0.60)	0.299
PT, %	99 (89–103)	105 (100–111)	107 (97–111)	106 (98–109)	105 (97–112)	0.403
APTT, s	25.0 (23.6–26.2)	25.8 (25.0–26.9)	26.0 (25.2–27.3)	24.2 (23.7–27.0)	26.0 (25.2–27.4)	0.249
Fibrinogen, g/L	2.8 (2.4–3.6)	2.7 (2.5–3.2)	2.7 (2.6–3.6)	2.8 (2.5–3.0)	3.2 (2.4–3.4)	0.791
D-dimer, µg/L FEU	264 (219–289)	248 (181–325)	227 (169–300)	288 (169–396)	361 (174–745)	0.598
BMI - Body Mass Index calculated as weight (kg)/height (cm)2. MET - measure of physical activity (min). WBC – white blood cell count. CRP - C-reactive protein. PT – prothrombin time. APTT – activated partial thromboplastin time. N – number of participant. All measurements are given as median (interquartile range, IQR), except age which is given as median (min – max). P < 0.05 is considered significant.						

The RIs for all measured parameters were derived and compared to the common national RIs, and the adopted manufacturer’s cut-off for D-dimers (Table 2). To be able to compare the statistically derived RI for WBC with the available common national RI, the total sample was divided into two subgroups according to their age. One group consisted of female athletes younger than 20 years old, and the other one comprised older athletes (≥ 20 years old), as common national RIs for WBC discriminate between those two groups (4.4–11.6 *vs.* 3.4–9.7 x10^9^/L). However, we were able to confirm the applicability of the common national RIs for WBC in both groups of young female athletes at rest. RI for PT was 80-119%, and this was the only RI for coagulation screening biomarkers which was in accordance with the common national RI (70–120%). The RI for aPTT in seconds was slightly lower (21.8–30.7 s) than the available manufacturer’s RI of 23.0–31.9 s. However, when we have calculated the aPTT-ratio, the common national RI has been confirmed (0.8–1.1 *vs.* 0.8–1.2). For Fbg the calculated RI was somewhat higher (1.9–4.4 g/L) than the common national RI of 1.8–3.5 g/L. D-dimer cut-off value was set at 852 µg/L FEU, higher than the proposed manufacturer’s cut-off of 500 µg/L FEU for deep vein thrombosis/pulmonary embolism (DVT/PE) diagnosis exclusion (*22*).

**Table 2:** Verifying the applicability of the common national reference intervals for inflammatory and coagulation biomarkers in a group of young healthy female athletes (N = 115)

**Analyte**	**Common national reference** **interval/cut-off value**		**Calculated reference ** **interval/cut-off value ** **for young female athletes**	**Common national reference interval/cut-off value verified (YES/NO)**
WBC, 10^9^/L	16–19 yrs	4.4–11.6	4.9–11.6	YES
	≥ 20 yrs	3.4–9.7	3.7–9.4	YES
CRP, mg/L	< 5.0		< 2.9	NO
PT, %	70–120		80–119	YES
APTT-ratio	0.8–1.2		0.8–1.1	YES
Fibrinogen, g/L	1.8–3.5		1.9–4.4	NO
D-dimer, µg/L FEU	< 500		< 852	NO
WBC – white blood cell count. CRP - C-reactive protein. PT – prothrombin time. APTT – activated partial thromboplastin time. N – number of participants.				

To explore the potential causality in observed RI differences, possible differences in measured variables between the activity subgroups were tested (Table 3). The statistically significant difference was observed between subgroups of low grade and moderate grade physical activity, and low-grade and high-grade physical activity in fibrinogen and D-dimer values, fibrinogen being higher in low-grade activity group (P = 0.021), while D-dimer being lower in the same group, compared to higher intensity groups (P = 0.008).

**TABLE 3:** Comparison of inflammatory and coagulation biomarkers in a group of young healthy female athletes, divided by their level of physical activity (N = 115)

**Analyte**	**Low grade physical** **activity (7/115, 6%)**	**Moderate grade physical** **activity (12/115, 10%)**	**High grade physical** **activity (96/115, 84%)**	**P**
WBC, 10^9^/L	5.9 (5.5–9.0)	6.3 (5.8–7.3)	6.4 (5.5–7.9)	0.971
CRP, mg/L	0.5 (0.4–1.8)	0.7 (0.5–1.2)	0.5 (0.3–0.9)	0.229
PT, %	102 (100–111)	106 (97–108)	105 (99–111)	0.999
APTT, s	25.2 (24.1–26.6)	25.5 (25.0–27.1)	25.9 (24.9–27.1)	0.638
Fibrinogen, g/L	3.5 (3.3–3.6)	2.7 (2.4–3.0)	2.7 (2.5–3.3)	0.021*
D-dimer, µg/L FEU	169 (169–175)	288 (238–440)	263 (180–335)	0.008*
WBC – white blood cell count. CRP – C-reactive protein. PT – prothrombin time. APTT – activated partial thromboplastin time. N – number of participants. All measurements are given as median (interquartile range, IQR). P < 0.05 is considered significant. *Significant difference between subgroups low grade and moderate grade physical activity, and low grade and high-grade physical activity.				

## Discussion

The most important finding of the study was that general, common national RIs for inflammatory and coagulation biomarkers are only partially applicable to the female athletes’ population. Metabolic and functional adaptations in an athlete’s body are substantially pronounced even at rest, resulting in biomarker’s concentrations being not comparable to the ones provided for general, healthy female population.

As previously reported, female athletes enrolled in this study did differ in age, weight, height, and, consequently, BMI, even after excluding six athletes who were OC-users (*14*). However, the athletes did not differ in their quantitative measure of physical activity expressed in metabolic equivalent (MET). That is why all sports have been considered as one sample and of all subject`s data was aggregated into one group, proceeding with the number large enough to investigate the applicability of the provided common national RIs for inflammatory and coagulation biomarkers.

Each exercise bout causes transient increases in total white blood cells, and a variety of plasma cytokines (*5*). It was documented that changes in circulating leukocyte numbers normally return to pre-existing values within 3–24h, and some cross-sectional studies reported very few differences in WBC count between sedentary individuals and athletes in the true resting state (*7*). Although acute phase proteins, including CRP, are also increased after heavy exertion, epidemiologic studies consistently show reduced inflammatory biomarkers (including WBC count and CRP) in adults with higher levels of physical activity and fitness (*23*). Those findings were also confirmed in the present study, which comprised of exclusively female athletes. WBCs at resting state were completely comparable with the common national available RIs derived from healthy non-athletes population; however, CRP concentrations in young athletes were lower than the proposed cut-off of 5.0 mg/L that discriminate between healthy individuals and inflammation. Our athletes exhibited CRP values at a lower cut-off of 2.9 mg/L (95^th^ percentile of the study group). This may lead to an unrecognized inflammatory process if their laboratory results are being managed in the same way as of the general population.

Regarding the coagulation biomarkers, the applicability of the available RIs was confirmed for PT activity and APTT-ratio. Although APTT values in seconds were in general shortened, the APTT-ratio confirmed the common national RI. However, fibrinogen concentrations were slightly increased compared to the general common national RIs. It was demonstrated before that exercise induces a significant increase in factor VIII activity which results in a significant shortening of aPTT (*23*-*25*). Regarding the fibrinogen activity, studies have given conflicting results, with some showing no difference after exercise, some showing an increase, and some showing a decrease (*26*-*28*). However, it has been postulated that fibrinogen levels are decreased after maximal and submaximal exercise, suggesting consumption during the coagulation cascade. Although our study results pointed to somewhat higher fibrinogen concentrations in female athletes at resting state, after dividing the group according to physical activity the fibrinogen concentration was significantly lower in subgroups of athletes that were physically more active than in the low activity subgroup. Consequently, D-dimers were higher in more physically active subgroups compared to the low activity subgroup. These findings support the claim that haemostatic balance is not maintained during recovery (*4*).

Whether the increased observed cut-off for D-dimers (< 852 µgl/L FEU) compared to the proposed cut-off of < 500 µg/L FEU for DVT/PE diagnosis exclusion points to a need for a higher cut-off, or just represents evidence for an increased hypercoagulability in athletes, remains unclear and should be further investigated. Nevertheless, in this study, none of the female athletes showed symptoms or reported any sign of the aforementioned diagnosis, neither at the time of the venepuncture nor within a year after that. The adopted cut-off has been confirmed on a Croatian healthy population by Coen Herak *et al*. with the same combination of an analyser and D-dimer reagent, as used in our study (*29*). The upper reference value determined in plasma samples from healthy volunteers was set at 495 µg/L FEU (*29*).

The limitations of this study should also not be omitted: the common national RIs were developed more than two decades ago, and it could be possible that they would be somewhat different if they had been updated since then, even in the general population; however, they are still in use in all MBLs in Croatia, which was the rationale for comparing the parameters measured in athletes with those available RIs. It is important to emphasize that the diagnosis of some disease generally is not based exclusively on the result of one test. Also, one pathological result outside the RIs does not immediately mean disease, as RIs cover only 95% of the population studied, implicating that 5% perfectly healthy individuals can have a pathological result. Nevertheless, it is evident that some common inflammatory and coagulation parameters were substantially different in our study group compared to the healthy non-athletes from which the national RIs have been derived, resulting in the non-applicability of the national common RIs.

In conclusion, the long-term chronic effects of exercise have an overall anti-inflammatory influence, leading to lower CRP concentrations compared to the general healthy population. However, a haemostatic balance is not maintained at rest, with lower observed fibrinogen concentrations in athletes with high physical activity level, compared to the subgroup of individuals with low physical activity level. Common national RIs derived from healthy non-athletes population cannot be applied for CRP and fibrinogen values in a group of healthy young athletes. A revision of available RIs is a must, taking into account exercise as a very significant covariate (*30*). A higher observed cut-off for D-dimers needs to be verified for DVT/PE diagnosis exclusion in a group of healthy young female athletes and should be taken into account when prescribing and following the effects of oral contraception on coagulation parameters. In general, the cut-off for D-dimer in VTE diagnostics should not be changed unless an extensive clinical study has priorly been performed.
